# Supplementary material for: Diagnosis of Pneumonia by Cough Sounds Analyzed with Statistical Features and AI
Source: Sensors (Basel). 2021 Oct 23;21(21):7036. doi: 10.3390/s21217036 (PMC8586978; doi:10.3390/s21217036)
Supplement: Supplementary file 1 [file sensors-21-07036-s001.zip › Supplementary Materials.pdf]

Title: Diagnosis of pneumonia by cough sounds analyzed with statistical feature and AI

Author: Youngbeen Chung[1], Jie Jin[5], Hyun In Jo[2], Hyun Lee[3], Sang-Heon Kim[3*], Sung Jun Chung[3], Ho Joo Yoon[3], Junhong Park[1**], Jin Yong Jeon[4]

[1] Department of Mechanical Engineering, Hanyang University, Wangsimni-ro 222, Seongdong-Gu, Seoul 04763, Republic of Korea

[2] Department of Architectural Engineering, Hanyang University, Wangsimni-ro 222, Seongdong-Gu, Seoul 04763, Republic of Korea

[3] Department of Internal Medicine, Hanyang University Hospital, Hanyang University College of Medicine, 222 Wangsimri-ro, Seongdong-gu, Seoul 04763, Republic of Korea

[4] Department of Medical and Digital Engineering, Hanyang University, Wangsimni-ro 222, Seongdong-Gu, Seoul 04763, Republic of Korea

[5] School of Electromechanical and Automotive Engineering, Yantai University, 30 Qingquan Road, Laishan District, Yantai, 264005, PR China

Corresponding author:

[*] Sang-Heon Kim: Department of Internal Medicine, Hanyang University College of Medicine, 222 Wangsimni-ro, Seongdong-gu, Seoul 04763, Republic of Korea

Tel: +82-02-2290-8336, Fax: +82-02-2298-9183, E-mail: sangheonkim@hanyang.ac.kr

[**] Junhong Park: Department of Mechanical Engineering, Hanyang University, 222 Wangsimni-ro, Seongdong-gu, Seoul 04673, Republic of Korea

Tel: +82-02-2220-0424, Fax: +82-02-2298-4634, E-mail: parkj@hanyang.ac.kr

Supplementary material:

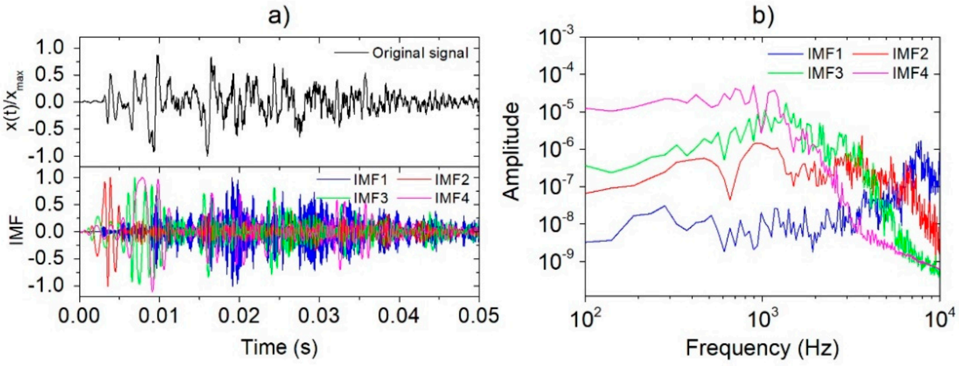

Supplementary Figure S1. Analysis of frequency ranges by components of cough sounds: a) IMF extraction according to frequency ranges with EMD method applied; b) peak frequency by power spectrum of each IMF.
