# Supplementary figures and images for: Diagnosis of Pneumonia by Cough Sounds Analyzed with Statistical Features and AI
Source: Sensors (Basel). 2021 Oct 23;21(21):7036. doi: 10.3390/s21217036 (PMC8586978; doi:10.3390/s21217036)

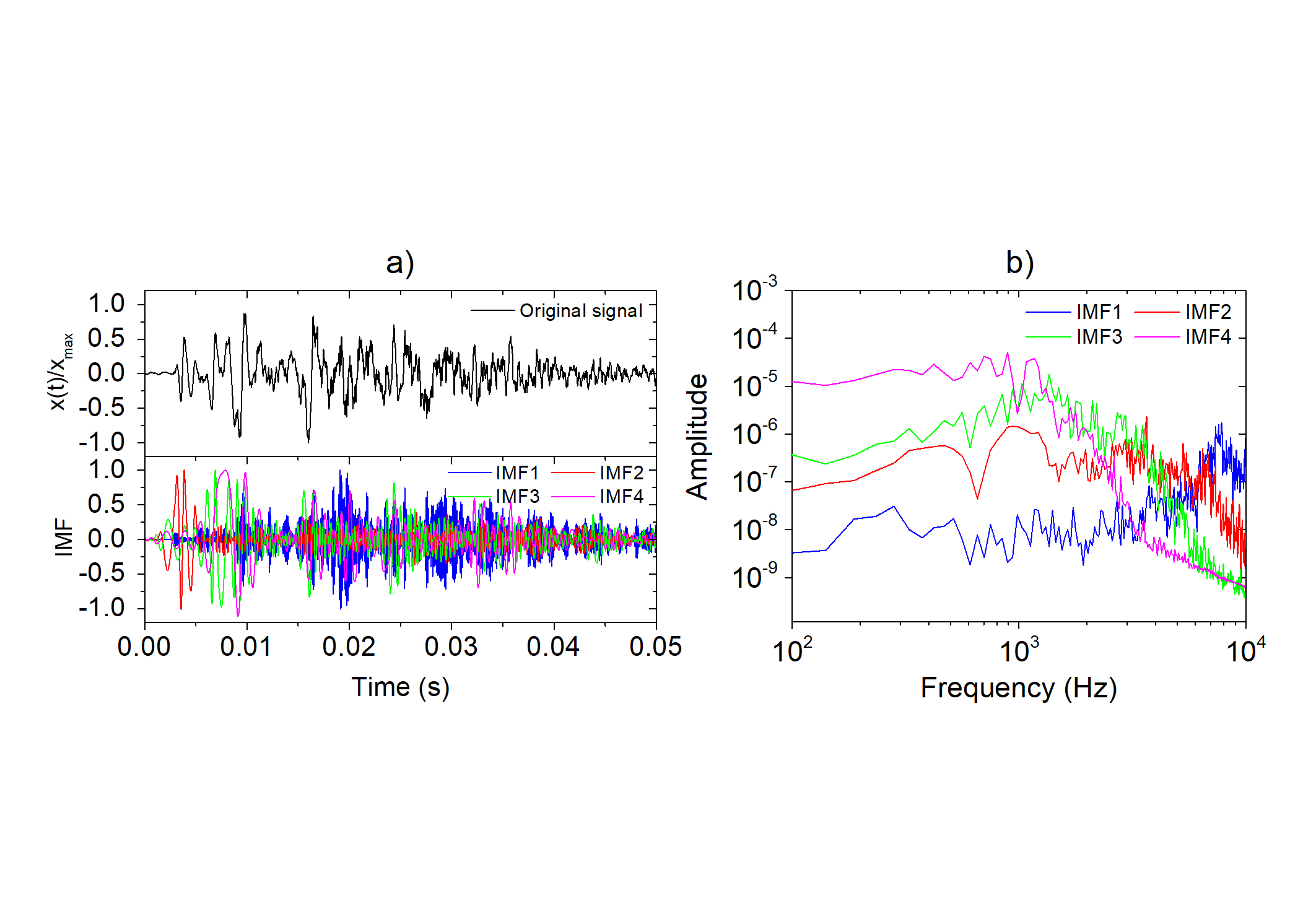

Supplement: Supplementary file 1 [file sensors-21-07036-s001.zip › Supplementary_Figure_S1.jpg]
